# An old friend: uric acid and its association with fractional flow reserve

**DOI:** 10.3906/sag-1903-91

**Published:** 2019-12-16

**Authors:** Salih TOPAL, Burak SEZENÖZ, Mustafa CANDEMİR, Eser AÇIKGÖZ, Sadık Kadri AÇIKGÖZ, Nuri Bülent BOYACI

**Affiliations:** 1 Department of Cardiology, Faculty of Medicine, Gazi University, Ankara Turkey; 2 Department of Cardiology, Yozgat State Hospital, Yozgat Turkey; 3 Department of Cardiology, Abdurrahman Yurtarslan Ankara Oncology Education and Research Hospital, Ankara Turkey; 4 Department of Cardiology, Kahraman Kazan Hamdi Eriş State Hospital, Kahramankazan Turkey

**Keywords:** Uric acid, fractional flow reserve, intermediate coronary stenosis

## Abstract

**Background/aim:**

The aim of this study was to investigate the importance of preprocedural uric acid (UA) level in predicting fractional flow reserve (FFR) results of intermediate coronary lesions in patients with stable coronary artery disease undergoing coronary angiography.

**Materials and methods:**

We retrospectively analyzed 293 patients who underwent FFR measurement to determine the significance of intermediate coronary stenosis detected by conventional coronary angiography. Patients were divided into 2 groups: Group 1 (n = 127) included patients with FFR of <0.80 (hemodynamically significant lesions), and Group 2 (n = 169) consisted of patients with FFR of >0.80 (hemodynamically nonsignificant lesions). Uric acid levels were assessed in both groups with the enzymatic colorimetric method by clinical chemistry autoanalyzer.

**Results:**

The mean UA level was significantly higher in patients whose FFR indicated hemodynamically significant coronary lesions (UA: 5.43 ± 1.29 mg/dL in Group 1 vs. 4.51 ± 1.34 mg/dL in Group 2, P < 0.001).

**Conclusion:**

Elevated UA levels are associated with hemodynamically significant coronary lesions measured with FFR. Uric acid may be used as a predictor of hemodynamically compromised coronary lesions before FFR procedures.

## 1. Introduction

Determining the functional significance of a coronary artery lesion is essential for revascularization decisions and prognosis estimation in patients with coronary artery disease (CAD). Coronary angiography (CAG) plays a crucial role in the assessment of coronary artery stenosis. However, it is inherently limited in its ability to demonstrate the functional significance of a coronary lesion, particularly in intermediate lesions, defined as those with 50%–70% stenosis [1]. Fractional flow reserve (FFR) is the most accurate method of determining the physiological significance of a coronary lesion and is therefore an important technique in daily cardiology practice [2]. 

Uric acid (UA) is an end-product of purine metabolism. There is growing evidence that elevated UA levels are correlated with both cardiovascular disease and its leading risk factors, such as hypertension [3]. Recent studies suggested that UA may be associated with endothelial dysfunction, oxidative stress, vasoconstriction, and inflammation, all of which are important in the development and progression of CAD [4,5]. Furthermore, UA elevation was shown to be positively correlated with CAD severity [6] and it may have a role in the prediction of prognosis after percutaneous coronary intervention in stable CAD [7]. Despite studies suggesting a link between UA and CAD, the relationship between UA and the functional significance of coronary lesions has yet to be determined. 

The aim of this study was to investigate the significance of preprocedural UA level as a predictor of FFR results in patients with stable CAD undergoing CAG.

## 2. Materials and methods

A total of 293 consecutive patients who underwent FFR measurement to determine the significance of intermediate coronary stenosis detected by conventional CAG were included in this retrospective study. Patients with acute coronary syndrome with or without positive cardiac markers, glomerular filtration rate of <60 mL/min, malignancy, hematological disorders, history of gout, ongoing treatment affecting UA levels such as allopurinol, chronic inflammatory diseases, or active infection were excluded from the study. Clinical risk factors such as age, sex, smoking status, diabetes mellitus (DM), hypertension, low-density lipoprotein (LDL) levels, and obesity were recorded. Written informed consent was obtained from each patient and the study was approved by the institutional ethics committee.

### 2.1. Laboratory testing

Blood samples were collected from the antecubital vein after 12 h of overnight fasting within 1 week before the procedure. Routine biochemistry and hemogram parameters were obtained. Uric acid levels were measured with the enzymatic colorimetric method by a clinical chemistry autoanalyzer (Aeroset, Abbott Laboratory, Abbott Park, IL, USA).

### 2.2. Coronary angiography and fractional flow reserve

The standard Judkins technique via femoral approach was used for coronary artery visualization. All patients were referred for elective coronary artery angiography by their attending physicians, who were blind to the study’s aim. Coronary angiography was performed using the DFP-8000D Toshiba digital radiography system. Intermediate coronary stenosis was defined as 50%–70% stenosis in any epicardial coronary artery (i.e. left anterior descending, circumflex, or right coronary artery). The SYNTAX I score was calculated using the SYNTAX score website (http://www.syntaxscore.com) by 2 interventional cardiologists who were blinded to the patients and each other. Measurement of fractional flow reserve was performed with a Radi 0.014 XT PW pressure-monitoring guidewire. After the pressure guidewire was calibrated and positioned distal to the lesion, an intracoronary adenosine bolus (initially 150 µg for the left coronary system vs. 100 µg for the right coronary artery) was administered to induce maximal vasodilatation by successively increasing the adenosine dose (maximum 600 µg) until no further decrement in FFR value was observed. Fractional flow reserve values were calculated as the ratio of mean distal coronary artery pressure to aortic pressure during basal and maximal vasodilatation. An FFR value of <0.80 after maximal hyperemia with adenosine was defined as hemodynamically significant. ∆FFR was measured using the following formula: FFR_rest_ − FFR_hyperemia_.

### 2.3. Statistical analysis

SPSS 21.0 for Windows (IBM Corp., Armonk, NY, USA) was used in all statistical analyses. The variables were investigated using visual (histograms, probability plots) and analytical (Kolmogorov–Smirnov test) methods to determine whether they were normally distributed. Descriptive analyses were presented using means and standard deviations for normally distributed variables and medians and interquartile range for nonnormally distributed variables. Categorical variables were presented as numbers and percentages. Student’s t-test or the Mann–Whitney U test were used to compare continuous variables, while the chi-square test and Fisher’s exact test, if necessary, were used to identify statistical differences for categorical variables. Pearson’s correlation analysis was used to assess correlations between UA and other parameters. Stepwise multivariate logistic regression analysis was done to examine the association between the functional significance of the lesions and other variables. Variables with P < 0.25 in univariate logistic regression were included in a multivariate logistic regression model. In the logistic regression model, UA level was assumed to be a binary variable according to the cutoff point detected in the receiver operating characteristic (ROC) curve analysis. P *< *0.05 was defined as statistically significant.

## 3. Results

Baseline characteristic features are shown in Table 1. The 293 patients in the study were divided into two groups: those with FFR values of <0.80 (Group 1, n = 127) and those with FFR values of ≥0.80 (Group 2, n = 169) after adenosine infusion. The mean age of the participants was 61.5 ± 9.3 years and 65.5% were male. The groups were similar in terms of age, sex, and prevalence of DM, hypertension, and smoking status. The mean UA level was significantly higher in group 1 (5.43 ± 1.29 vs. 4.51 ± 1.34 mg/dL, P < 0.001). The lactose dehydrogenase (LDH) level was also significantly higher in Group 1, whereas red blood cell (RBC) count and hemoglobin (Hb) level were higher in Group 2. 

**Table 1:** Baseline characteristic features.

	Total (n = 293)	Group 1 (n = 126)	Group 2 (n = 167)	P-value
Age, years	61.5 ± 9.3	61.1 ± 9	62 ± 9.5	0.471
Sex, male, n (%)	192 (65.5)	90 (71.4)	102 (61)	0.085
Hypertension, n (%)	136 (46.4)	60 (47.6)	76 (45.5)	0.810
Diabetes mellitus, n (%)	114 (38.9)	54 (42.9)	60 (35.9)	0.279
Smoking, n (%)	129 (44.0)	55 (43.7)	74 (44.3)	0.910
BUN, mg/dL	16 (14–20)	16 (14–20)	16.5 (14–20)	0.918
Creatine, mg/dL	0.9 (0.8–1.1)	0.9 (0.8–1.1)	0.9 (0.8–1.1)	0.913
Glucose, mg/dL	111 (95–154)	115 (95–163)	109 (94–146)	0.322
AST, U/L	20 (17–27)	20 (16–26)	21 (17–27)	0.477
ALT, U/L	21 (15–28)	20 (15–29)	21 (15–28)	0.460
Uric acid, mg/dL	4.9 ± 1.4	5.4 ± 1.3	4.5 ± 1.3	<0.001
LDH, U/L	192 (163–225)	204 (170–228)	182 (159–225)	0.048
GGT, U/L	36 (22–48)	36 (25–50)	36 (21–43)	0.095
WBC, ×10^3^/mL	8.2 ± 6.6	8.1 ± 2.4	8.3 ± 2.4	0.680
RBC, ×10^6^/mL	4.6 ± 0.6	4.5 ± 1	4.6 ± 0.6	0.036
Hemoglobin, g/dL	13.6 ± 2.3	13.3 ± 2.1	13.8 ± 2.5	0.049
MCV, fL	88 (85–91)	88 (85–91)	87 (85–91)	0.371
RDW, %	13.8 (13–15)	13.7 (13–15)	13.8 (13–15)	0.792
Platelets, ×10^3^/mL	240 ± 67.5	241 ± 73	239 ± 63	0.745
MPV, fL	8.7 (8–9.5)	8.6 (7.9–9.5)	8.8 (8–9.6)	0.353
PDW, %	16.6 (16–17)	16.6 (16–17)	16.6 (16–17)	0.170
Total cholesterol, mg/dL	190 ± 54	192 ± 54	188 ± 54	0.497
LDL, mg/dL	118 ± 46	121 ± 45	116 ± 46	0.358
HDL, mg/dL	40 (34–47)	38 (34–46)	41 (34–48)	0.154
Triglyceride, mg/dL	134 (92–207)	136 (97–202)	128 (87–214)	0.713
SYNTAX I score	12.5 ± 7.2	10.8 ± 6.6	14.9 ± 7.5	0.182

BUN, Blood urea nitrogen; AST, aspartate transaminase; ALT, alanine transaminase; LDH, lactate dehydrogenase; GGT, gamma-glutamyl transferase; WBC, white blood cells; RBC, red blood cells; MCV, mean corpuscular volume’ RDW, red cell distribution width; MPV, mean platelet volume; PDW, platelet distribution width; LDL, low-density lipoprotein; HDL, high-density lipoprotein.

There was no significant correlation between UA level and SYNTAX I score (P = 0.136, r = 0.087). Baseline FFR before adenosine was significantly lower in Group 1 than Group 2 (0.87 ± 0.04 vs. 0.93 ± 0.03, P < 0.001). There was a weak but significant positive correlation between UA level and ∆FFR (r = 0.221, P < 0.001) (Table 2). ROC curve analysis revealed that a UA cutoff point of 4.95 mg/dL had 65.1% sensitivity and 66.5% specificity in detecting significant functional stenosis in FFR measurements (Figure). When the patients were grouped according to this cutoff level, the group with high UA had significantly lower FFR values at baseline and maximal hyperemia and significantly greater ∆FFR (Table 2). In multivariate logistic regression analysis, UA (OR 3.970, CI: 2.383–6.643, P < 0.001) and RBC count (OR 0.999, CI: 0.999–1.000, P = 0.007) were identified as independent predictors of significant functional stenosis (Table 3). 

**Figure 1:**
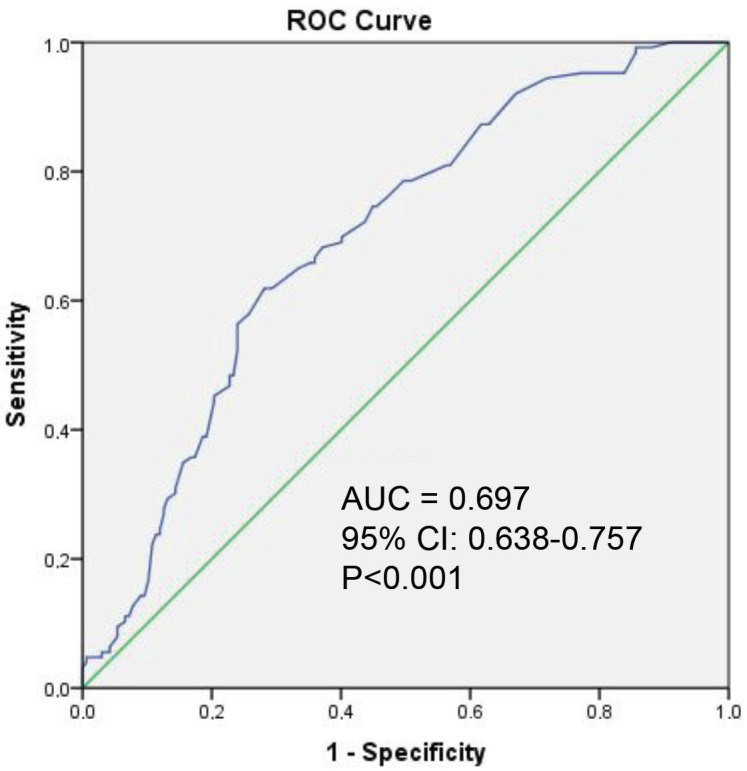
Receiver operating characteristic (ROC) curve analysis of uric acid level for the detection of significant functional stenosis in FFR measurements.

**Table 2:** Comparison of fractional flow reserve (FFR) values between the low and high uric acid groups.

	Uric acid < 4.95 mg/dL	Uric acid ≥ 4.95 mg/dL	P-value
FFR, baseline	0.91 ± 0.05	0.89 ± 0.05	0.002
FFR, after adenosine	0.82 ± 0.07	0.77 ± 0.08	<0.001
∆FFR	0.09 ± 0.05	0.12 ± 0.06	< 0.001

FFR, Fractional flow reserve.

**Table 3:** Factors predicting functional significance in multivariate logistic regression analysis.

	Univariate		Multivariate	
Variables	OR (95% CI)	P-value	OR (95% CI)	P-value
Age	0.969 (0.940–0.999)	0.046	0.975 (0.948–1.002)	0.073
Male sex	1.557 (0.862–2.811)	0.142	1.606 (0.932–2.767)	0.088
Hypertension	1.308 (0.752–2.273)	0.342	-	-
Diabetes mellitus	1.145 (0.671–1.955)	0.620	-	-
Hemoglobin	0.963 (0.790–1.173)	0.707	-	-
RBC	1.000 (0.999–1.000)	0.181	0.999 (0.999–1.000)	0.007
PDW	1.115 (0.927–1.342)	0.253	-	-
LDH	1.004 (1.000–1.008)	0.069	1.004 (1.000–1.008)	0.066
Uric acid > 4.95 mg/dL	3.738 (2.215–6.306)	<0.001	3.979 (2.383–6.643)	<0.001
HDL	0.989 (0.963–1.016)	0.425	-	-

RBC, Red blood cell; PDW, platelet distribution width; LDH, lactate dehydrogenase; HDL, high-density lipoprotein.

## 4. Discussion

The main finding of the present study is that admission UA level is significantly and independently associated with the functional significance of angiographically intermediate coronary stenosis. To the best of our knowledge, this is the first study to report such a relationship. Although RBC count was the other independent predictor of functional stenosis, an odds ratio of 0.999 means that this association is unlikely to be of clinical importance. 

Intermediate stenosis (50%–70%) is a common finding in CAG, and deciding whether to pursue medical or interventional treatment is difficult for such lesions. Fractional flow reserve is the gold-standard method for decision-making in intermediate lesions because it provides precise information about physiological hemodynamic status rather than a prediction from anatomical appearance [8]. On the other hand, FFR is an invasive method with some cost, and more easily attainable, cheaper, and repeatable markers would be welcomed. Thus, UA may be a valuable marker for both diagnosis and treatment decisions.

Studies have revealed that hyperuricemia is associated with hypertension, atherosclerosis, and even sudden cardiac death, but its exact relationship with cardiovascular outcomes remains controversial [9]. Previous studies showed that high UA level is associated with more extensive and severe CAD, poorer coronary collateral circulation, and more coronary calcification, all of which are related to poor prognosis [10]. Ndrepepa et al. also showed that higher UA level was a predictor of increased risk of mortality in 13,723 patients with angiography-proven CAD [11]. 

The role of uric acid in the formation and progression of CAD is not well established. However, it was shown that UA has some mechanical and molecular effects such as promotion of inflammation, vasoconstriction, and endothelial dysfunction, which may contribute to atherosclerosis and related comorbidities such as CAD. Moreover, hyperuricemia induces oxidative stress by an unknown mechanism. Antioxidant treatment in hyperuricemic rats resulted in hypertension remission and improved proinflammatory effects [5,12]. Uric acid uptaken by cells stimulates chemokine and inflammatory marker synthesis, which also leads to activation of vasoconstrictor mediators such as thromboxane, endothelin-1, and angiotensin-II [13,14]. 

Our data showed that a preprocedurally elevated UA level is associated with hemodynamically significant lesions measured by FFR during index angiography. This result is compatible with previous studies that also reported its correlation with CAD presence and impaired myocardial perfusion [15,16]. 

The above-mentioned vasoconstrictor mechanism in hyperuricemia and its role in atherosclerosis pathogenesis results in impaired vasorelaxation. Inducing hyperemia is a key part of the FFR procedure, and hyperuricemia may affect adenosine-related vasodilation. Higher ∆FFR values may be attributable to a reversal of baseline hyperuricemia-induced vasoconstriction by adenosine administration. Hyperuricemia impairs endothelial function in various ways, especially endothelial-dependent vasodilation, thus promoting vasoconstriction. Moreover, as adenosine enhances NO release from the endothelium, endothelial dysfunction will lead to impaired response to adenosine challenge, which is also a possible contributor to lower FFR values. Coronary artery disease causing ischemia eventually results in ATP reduction in the myocardium. Studies have shown that ATP depletion may be an important contributor to uric acid overproduction [17], which in turn increases apoptosis, oxidative stress, and a jeopardized myocardium [18]. We suppose that the relationship between ATP and uric acid level needs to be investigated.

Patients with FFR values in the gray zone (0.75–0.80) are the most complicated cases for determining lesion severity, and thus the most challenging in terms of treatment decisions. For these patients, Kocaman et al. proposed using ∆FFR, which is defined as the difference between resting FFR and FFR after adenosine administration, and suggested that ∆FFR represents the compensatory response capacity of the coronary microvasculature [19]. In the present study, higher ∆FFR values were detected in patients with higher UA levels. Thus, higher UA levels may be an indicator of severe lesions in the gray zone.

Previous studies have shown positive correlations between SYNTAX scores and serum uric acid levels, unlike our study. However, our study involved a population more likely to have type A lesions and low SYNTAX scores. These findings may be responsible for this unexpected result.

Numerous factors influence atherosclerosis and coronary artery lesion severity. In our patients groups, the distribution of risk factors and other biochemical markers (fasting glucose, HbA1c level, creatinine) were similar. These well-matched groups strengthened the power of our study, ensuring that UA was the only biochemical and risk factor difference.

In conclusion, elevated UA level is associated with hemodynamically significant coronary lesions in FFR. A significant but weak positive correlation was detected between UA levels and ∆FFR values. UA may have a role in the prediction of hemodynamically significant coronary lesions; however, further studies are needed to validate this finding in large-scale trials.

### 4.1. Study limitations

This study has some limitations. First of all, the number of patients is a major disadvantage and limits the statistical power of the study. Second, antihypertensive and antidiabetic medications may influence UA levels. Other medications affecting endothelial function (statins, ACE inhibitors) and uric acid level (thiazide diuretics) may also have a role in vascular response. Third, it is more plausible to conduct a prospective study to investigate the direct effect of UA during adenosine administration. Single-center trials may not reflect a nationwide and generalized outcome.
